# Aggressiveness in Well-Differentiated Small Intestinal Neuroendocrine Tumors: A Rare Case and Narrative Literature Review

**DOI:** 10.3390/jcm14165821

**Published:** 2025-08-18

**Authors:** Laurențiu Augustus Barbu, Liviu Vasile, Liliana Cercelaru, Valeriu Șurlin, Stelian-Ștefaniță Mogoantă, Gabriel Florin Răzvan Mogoș, Tiberiu Stefăniță Țenea Cojan, Nicolae-Dragoș Mărgăritescu, Marius P. Iordache, Anca Buliman

**Affiliations:** 1 Department of Surgery, Railway Clinical Hospital Craiova, University of Medicine and Pharmacy of Craiova, 2 Petru Rares Street, 200349 Craiova, Romania; laurentiu.barbu@umfcv.ro (L.A.B.); gabriel.mogos@umfcv.ro (G.F.R.M.); tiberiu.tenea@umfcv.ro (T.S.Ț.C.); 2 Department of Surgery, Emergency County Hospital, University of Medicine and Pharmacy of Craiova, 2 Petru Rares Street, 200349 Craiova, Romania; vliviu777@yahoo.com (L.V.); vsurlin@gmail.com (V.Ș.); ssmogo@yahoo.com (S.-Ș.M.); dmargaritescu@yahoo.com (N.-D.M.); 3 Department of Embryology and Anatomy, University of Medicine and Pharmacy of Craiova, 200349 Craiova, Romania; liliana.cercelaru@umfcv.ro; 4 Department of Clinical Neurology, Faculty of Medicine, Titu Maiorescu University, 67A Gheorghe Petrașcu Street, 031593 Bucharest, Romania; 5 Department of Radiology, Faculty of Medicine, Titu Maiorescu University, 67A Gheorghe Petrașcu Street, 031593 Bucharest, Romania; buliman_anca@yahoo.com

**Keywords:** small intestinal neuroendocrine tumor, SI-NET, carcinoid, peritoneal metastasis, liver metastasis, chromogranin A, PRRT, immunohistochemistry

## Abstract

**Background:** Small intestinal neuroendocrine tumors (SI-NETs) are the most common malignancies of the small bowel. Although typically well differentiated and slow-growing, they may exhibit aggressive behavior, especially when diagnosed at an advanced stage. **Objective:** To illustrate the diagnostic and therapeutic challenges of advanced SI-NETs through a rare case presentation and a narrative review of recent studies in the literature. **Methods:** A narrative literature review was conducted using the PubMed database to examine the incidence, risk factors, diagnostic modalities, and treatment strategies for advanced-stage SI-NETs. The search included studies published between January 2010 and June 2025 and focused on human subjects, using keywords such as “small intestinal neuroendocrine tumor”, “metastasis”, “tumor grade”, and “treatment”. **Results:** We report the case of a 68-year-old man who presented with bowel obstruction. Imaging and surgical exploration revealed a jejunoileal SI-NET with extensive liver and peritoneal metastases, mesenteric fibrosis, and ascites. Histopathology confirmed a well-differentiated grade 2 tumor (Ki-67: 3%) positive for chromogranin A and CD56. Despite a low proliferative index, the tumor demonstrated aggressive clinical behavior. The patient underwent emergency enterectomy with ileostomy and was referred for further evaluation, including somatostatin receptor imaging and consideration for peptide receptor radionuclide therapy (PRRT). **Conclusions:** This case highlights the potential for aggressive progression in well-differentiated SI-NETs with low Ki-67 indices. Histological grade alone may not predict clinical behavior. Early diagnosis, comprehensive staging, and individualized multidisciplinary management—guided by functional imaging and receptor profiling—are critical to improving outcomes in advanced SI-NETs.

## 1. Introduction

Small intestinal neuroendocrine tumors (SI-NETs) are the most common malignancies of the small bowel, with their incidence steadily increasing over recent decades [1,2,3]. Due to their small size, submucosal location, and often asymptomatic presentation, these tumors are frequently diagnosed at advanced stages, with up to 50% of cases presenting with distant metastases at the time of diagnosis [4,5,6,7]. Although well-differentiated SI-NETs are typically considered indolent, clinical behavior can vary significantly, and aggressive progression is not uncommon—even in tumors with low proliferative indices [8,9,10,11].

These neoplasms are characterized by neuroendocrine marker expression, such as chromogranin A and synaptophysin, and are graded based on mitotic count and Ki-67 index [12]. Histological classification, while important, does not always correlate with clinical outcomes, emphasizing the need for comprehensive diagnostic and therapeutic approaches [13].

In recent years, efforts by groups such as the European Neuroendocrine Tumor Society (ENETS) have led to the adoption of standardized grading and staging systems to improve prognostic accuracy and guide treatment planning [14,15].

Here, we report a rare and clinically complex case of a jejunoileal SI-NET presenting with extensive hepatic and peritoneal metastases and bowel obstruction, highlighting key challenges in diagnosis and management. This case is complemented by a narrative review of the literature to contextualize its implications.

## 2. Literature Review Methodology

A narrative literature review was conducted using the PubMed database to examine the incidence, risk factors, diagnostic tools, and treatment strategies for advanced-stage small intestinal neuroendocrine tumors (SI-NETs), with an emphasis on the relationship between tumor grade, metastatic spread, and clinical behavior. The search was performed using combinations of keywords such as “small intestinal neuroendocrine tumor”, “advanced stage”, “metastasis”, “tumor grade”, “diagnosis”, and “treatment”. The search covered studies in the literature published between January 2010 and June 2025.

Inclusion criteria were as follows: peer-reviewed articles in English, focused on human subjects, addressing clinical aspects of SI-NETs at advanced stages. Exclusion criteria included case reports, non-English publications, animal studies, and articles not directly addressing tumor staging or metastasis.

## 3. Case Presentation

### 3.1. Patient Information

A 68-year-old male of Romanian ethnicity, with no significant prior history of gastrointestinal disease or known malignancy, presented to the Emergency County Hospital Slatina. The patient’s medical history included arterial hypertension well controlled with medication. He had no family history of neuroendocrine tumors, gastrointestinal cancers, or inherited syndromes (e.g., MEN1, NF1). He was a non-smoker and consumed alcohol occasionally. No relevant occupational exposures were identified.

### 3.2. Presenting Concerns

The patient reported a 7-day history of progressive diffuse abdominal pain, associated with cessation of stool and gas passage, and multiple episodes of non-bilious vomiting. He also complained of progressive abdominal distension and mild weight loss over the preceding 2 months, which had not been previously investigated.

### 3.3. Clinical Findings

On admission, the patient was hemodynamically stable but appeared mildly dehydrated. Physical examination revealed a markedly distended abdomen with diffuse tenderness on palpation. No peritoneal signs were evident (i.e., no guarding or rebound). Bowel sounds were hyperactive. Digital rectal examination revealed an empty rectum. There was no evidence of palpable peripheral lymphadenopathy or hepatosplenomegaly.

### 3.4. Diagnostic Assessment

**Figure 1:**
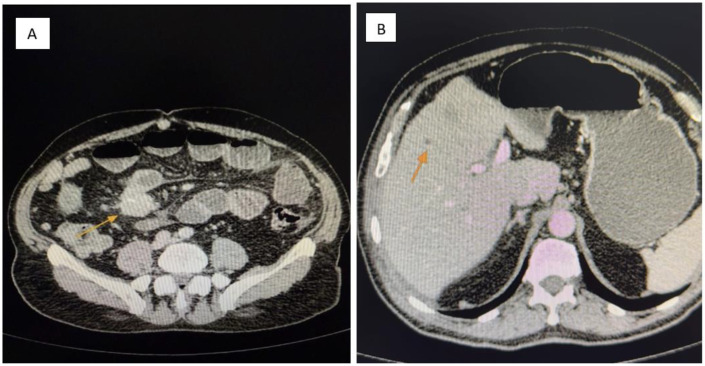
(**A,B**): **Contrast-enhanced abdominal CT.** (**A**) A pelvic mesenteric tumor mass (yellow arrow) is seen causing significant dilatation of adjacent small bowel loops, suggestive of partial obstruction. (**B**) Multiple hypodense hepatic lesions (yellow arrow) are visualized, consistent with secondary metastatic deposits from a neuroendocrine tumor.

**Figure 2:**
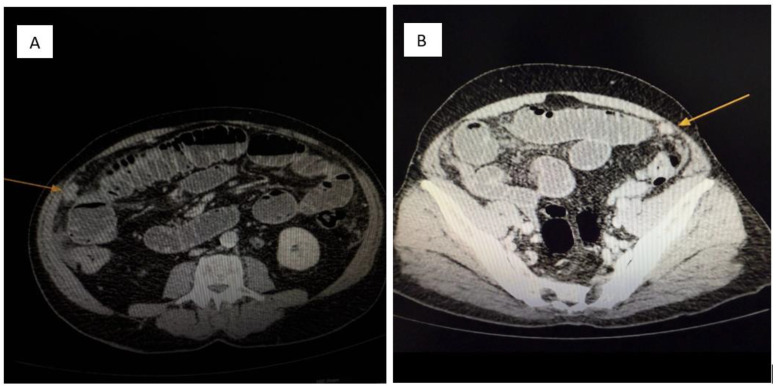
(**A**,**B**): **Contrast-enhanced abdominal CT.** Multiple soft-tissue density nodules (yellow arrow) are diffusely distributed throughout the peritoneal cavity, consistent with metastatic involvement of the parietal peritoneum. The pattern and morphology of these lesions support a diagnosis of peritoneal carcinomatosis (yellow arrow) secondary to a neuroendocrine tumor.

Laboratory investigations showed leukocytosis (WBC 18,000/mm^3^), elevated C-reactive protein (CRP 65 mg/L), mild electrolyte imbalance (hypokalemia, hyponatremia), and moderate anemia (Hb 10.2 g/dL). Liver function tests and renal function were within normal limits. Tumor markers CEA and CA 19-9 were not elevated at admission but chromogranin A was not available emergently. Contrast-enhanced abdominal and pelvic CT (Siemens Healthineers, Erlangen, Germany) revealed the following:

- A 5 cm mesenteric mass in the pelvic region (Figure 1A).
- Multiple hypodense hepatic nodules compatible with liver metastases (Figure 1B).
- Markedly dilated loops of jejunum and ileum with wall thickening and multiple air–fluid levels.
- Significant ascites and diffuse peritoneal nodular thickening suggestive of carcinomatosis (Figure 2).

### 3.5. Therapeutic Intervention

**Figure 3:**
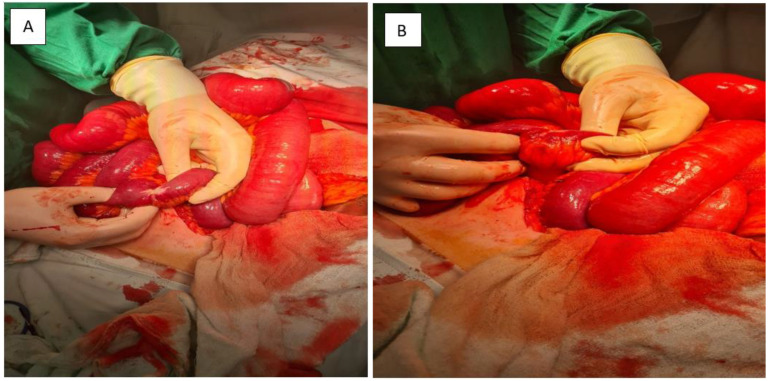
(**A,B**): **Intraoperative view.** A markedly stenotic segment of small intestine is shown, with significant proximal bowel dilatation. These findings are consistent with partial obstruction secondary to tumor infiltration and associated mesenteric fibrosis.

After multidisciplinary emergency discussion, surgical exploration was indicated due to signs of intestinal obstruction. Under general anesthesia, a midline laparotomy was performed. Intraoperative findings confirmed the following:

- Approximately 2 L of straw-colored ascites.
- Multiple whitish tumor deposits on the serosal surfaces of the small bowel, omentum, and peritoneum (Figure 3).
- A palpable, irregular mesenteric mass infiltrating the mesentery and sigmoid colon with significant mesenteric retraction and dense fibrosis.
- Multiple omental nodules (up to 3 cm) and hepatic metastases on segments II and III.

A segment of stenotic small bowel was resected and a terminal ileostomy was created approximately 160 cm from the duodenojejunal flexure, exteriorized in the right iliac fossa. Representative omental and peritoneal nodules were excised for histopathological diagnosis.

### 3.6. Postoperative Course and Follow-Up

The patient’s immediate postoperative course was uneventful. Bowel function resumed progressively, oral feeding was reintroduced, and no complications such as wound infection or anastomotic leakage occurred. Histopathological examination of the resected specimens confirmed a well-differentiated (G2) neuroendocrine tumor (NET) with a Ki-67 index of approximately 3%. Immunohistochemical staining showed strong diffuse positivity for chromogranin A and CD56, and focal weak positivity for synaptophysin. No tumor necrosis was observed. Final staging was consistent with advanced disease (AJCC stage IV) due to synchronous peritoneal carcinomatosis and liver metastases.

The patient was discharged in good general condition on postoperative day 9, with referral to the institutional tumor board for further management. A follow-up plan was agreed, including additional functional imaging with somatostatin receptor scintigraphy (SRS) or Ga-68 PET/CT, and evaluation for peptide receptor radionuclide therapy (PRRT). The patient continues to be monitored regularly by a multidisciplinary team including surgery, oncology, nuclear medicine, and gastroenterology.

### 3.7. Patient Perspective

The patient expressed gratitude for the prompt emergency intervention, stating that the resolution of obstructive symptoms significantly improved his quality of life. He provided full written informed consent for the publication of his clinical details and images for scientific purposes.

### 3.8. Informed Consent

Written informed consent was obtained from the patient for publication of this case report and any accompanying images. A copy of the written consent is available for review by the Editor-in-Chief of this journal on request.

**Histopathological Findings** All surgical specimens were processed using standard histopathological protocols.

**Figure 4:**
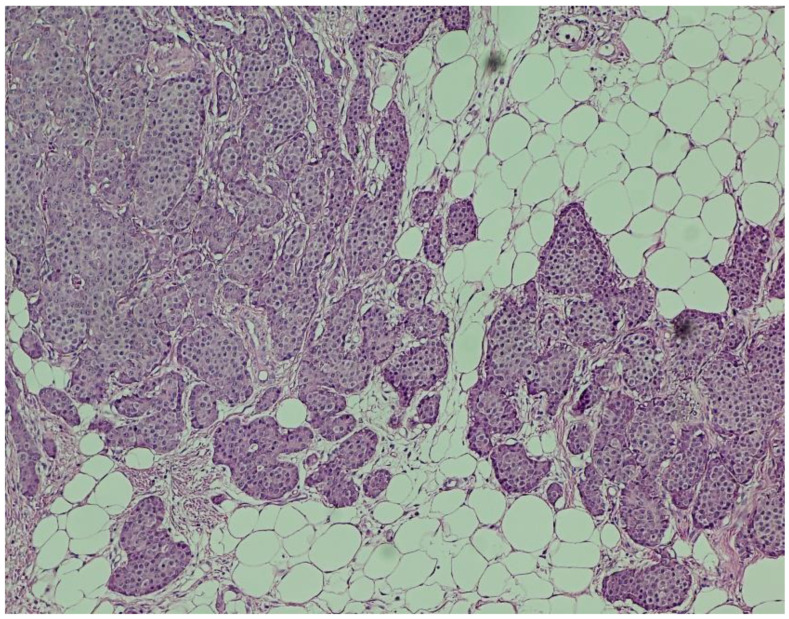
**Hematoxylin and eosin (H&E) staining (×100).** Nests of uniform neuroendocrine tumor cells exhibiting round nuclei, “salt and pepper” chromatin, and minimal cytological atypia, consistent with a well-differentiated morphology.

**Hematoxylin and eosin (H&E) staining** revealed a diffuse proliferation of tumor cells within adipose tissue, predominantly arranged in nested and insular patterns, with focal areas of trabecular architecture. The neoplastic cells were uniform in appearance, displaying round to oval nuclei, finely granular “salt-and-pepper” chromatin, and scant eosinophilic cytoplasm (Figure 4). Mitotic figures were rare (fewer than two per ten high-power fields), and no areas of tumor necrosis were identified—features consistent with a well-differentiated neuroendocrine tumor.

**Figure 5:**
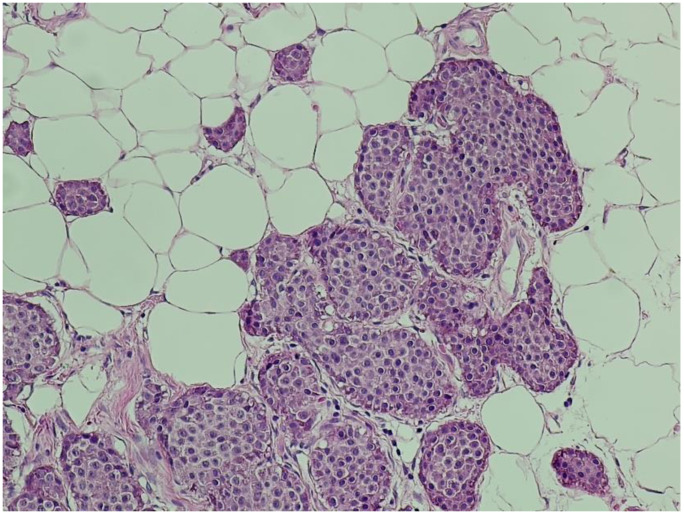
**Chromogranin A immunostaining (×100).** Diffuse and strong cytoplasmic staining is observed in nests of uniform neuroendocrine tumor cells, confirming neuroendocrine differentiation.

**Figure 6:**
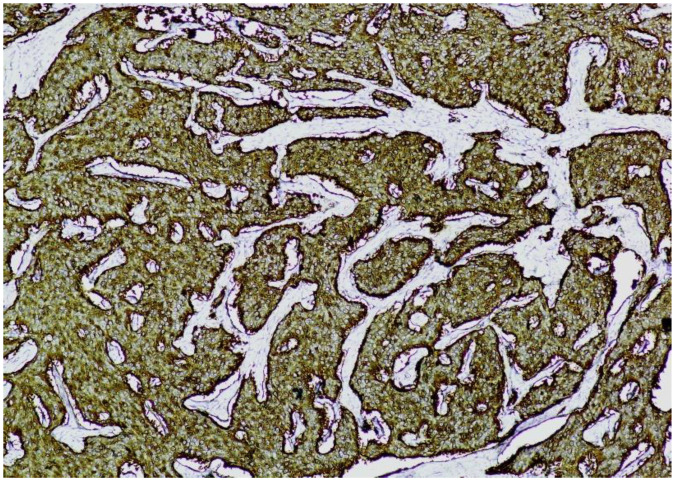
**Chromogranin A immunostaining (×100).** Strong and diffuse cytoplasmic positivity is observed in nests of uniform neuroendocrine tumor cells, consistent with neuroendocrine differentiation.

**Figure 7:**
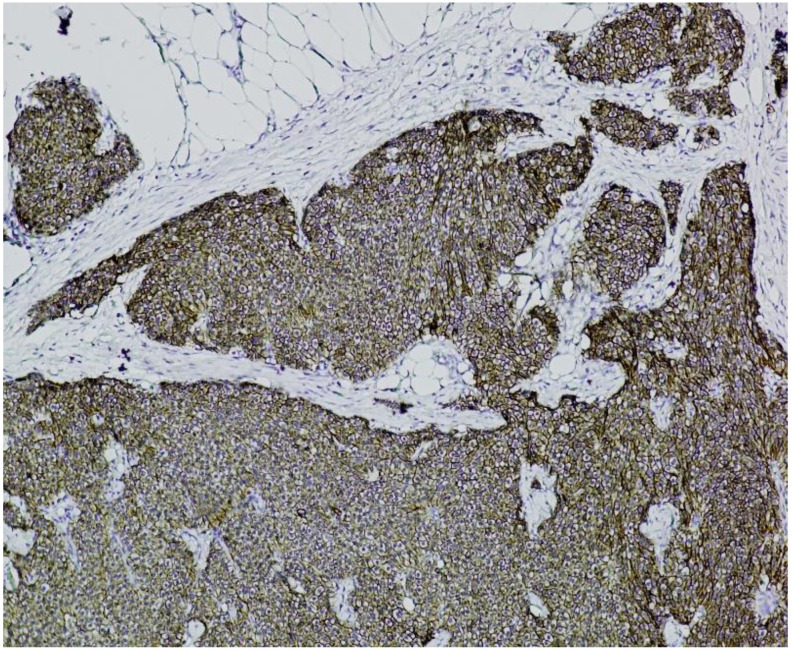
**CD56 immunostaining (×100).** The image shows nests of well-differentiated neuroendocrine tumor cells exhibiting diffuse and strong membranous CD56 positivity, consistent with neuroendocrine phenotype.

**Figure 8:**
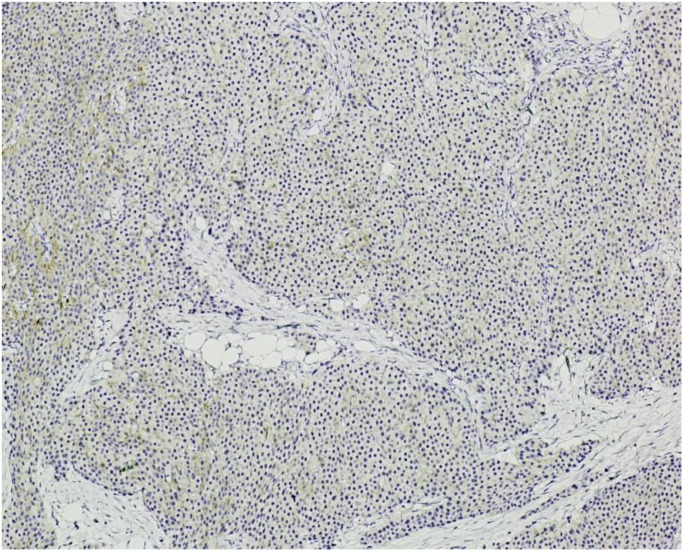
**Synaptophysin immunostaining, ×100.** Focal and weak immunoreactivity is observed in clusters of monotonous neuroendocrine cells, consistent with variable synaptophysin expression in well-differentiated neuroendocrine tumors.

**Immunohistochemistry (IHC)** showed diffuse and strong positivity for chromogranin A and CD56, with focal weak staining for synaptophysin, supporting neuroendocrine differentiation (Figure 5, Figure 6, Figure 7 and Figure 8).

The **Ki-67 proliferation index** was estimated at approximately 3%, corresponding to a **grade 2 (G2)** neuroendocrine tumor according to WHO classification.

Based on intraoperative findings and radiologic imaging, the tumor was staged as **stage IV** according to the **American Joint Committee on Cancer (AJCC)** staging system, due to the presence of both hepatic and peritoneal metastases.

## 4. Incidence and Diagnostic Challenges

Small intestinal neuroendocrine tumors (SI-NETs) represent the most frequent neoplasms of the small bowel, accounting for approximately 35% of small intestinal malignancies [16]. Their incidence has increased markedly over recent decades, with a threefold rise observed in the United States between 1973 and 2002 [16]. This trend underscores their growing clinical relevance and the need for improved diagnostic strategies.

The delayed diagnosis of SI-NETs is often attributed to their small size, submucosal growth, and frequently nonspecific or absent symptoms. A substantial proportion of patients are diagnosed at an advanced stage, often following complications such as bowel obstruction or gastrointestinal bleeding [17,18]. In one study, the average diagnostic delay exceeded four years, highlighting the indolent and deceptive course of these tumors [19].

Srirajaskanthan et al. emphasized the biological heterogeneity of these tumors and the importance of accurate staging systems, such as those proposed by the European Neuroendocrine Tumor Society (ENETS), to improve prognostic accuracy and guide treatment [15]. Neychev et al. further noted that while approximately 85% of gastroenteropancreatic NETs are sporadic, their clinical presentation varies widely depending on hormonal activity, with functional tumors often manifesting as carcinoid syndrome [16].

Nikiforchin et al. described the ileum as the most common site of origin and noted that nearly 50% of cases present with metastatic disease at diagnosis, including peritoneal carcinomatosis in up to 17% of cases [20]. Ahmed also reported on the wide biological spectrum of NETs—from indolent to aggressive—and reinforced the importance of individualized diagnostic workup and staging, particularly considering hereditary syndromes such as MEN1 and NF1 that may underlie some cases [21].

Taken together, these findings reflect the evolving understanding of SI-NETs as clinically diverse tumors whose indolent morphology may belie their aggressive potential. Diagnostic delay remains a significant challenge, reinforcing the need for increased clinical awareness, better imaging modalities, and integrated multidisciplinary evaluation.

## 5. Risk Factors and Etiology

Neuroendocrine tumors also known as carcinoid tumors, most commonly occur sporadically but may be associated with hereditary syndromes such as multiple endocrine neoplasia type 1 (MEN1), neurofibromatosis type 1 (NF1), von Hippel–Lindau disease (VHL), and, more rarely, tuberous sclerosis. Specific NET subtypes are linked to these syndromes: pancreatic, thymic, and bronchial NETs in MEN1; duodenal and periampullary NETs in NF1; and pancreatic NETs in VHL. Additionally, conditions that induce hypergastrinemia—such as chronic atrophic gastritis, pernicious anemia, and Zollinger–Ellison syndrome—are associated with an increased risk of gastric NETs.

NETs are most frequently diagnosed in adults aged 50 to 70 years, with a slight female predominance for gastrointestinal forms, whereas bronchial carcinoids appear to affect both sexes equally. Environmental risk factors are limited; however, smoking has been weakly associated with bronchial carcinoids [22]. The appendix is a frequent site in younger adults, often discovered incidentally, while midgut NETs—particularly those of the ileum—are most commonly associated with carcinoid syndrome, especially when hepatic metastases are present.

Although tobacco and alcohol use are not strongly associated with NETs, a high intake of saturated fats has demonstrated a significant correlation with tumor development [17,22].

Additionally, familial associations have been observed. Small bowel carcinoids appear more frequently in individuals with first-degree relatives diagnosed with prostate or colorectal cancer [23], and among those with siblings affected by oral cavity malignancies [24]. These findings support the potential role of genetic predisposition in a subset of patients with SI-NETs.

## 6. Morphological and Histological Features

Well-differentiated neuroendocrine tumors (NETs) exhibit uniform cellular morphology and characteristic organoid architectural patterns—such as trabecular, gyriform, or nested arrangements. These tumors contain numerous neurosecretory granules and consistently express immunohistochemical markers like chromogranin A and synaptophysin [25].

Tumor grade is a key prognostic factor, with well-differentiated NETs typically classified as low or intermediate grade, indicating relatively indolent behavior. In contrast, poorly differentiated neoplasms correspond to high-grade categories and are associated with aggressive clinical progression [25].

## 7. Imaging and Functional Diagnostics

Follow-up imaging after complete surgical resection is recommended within 6 to 12 months, even in patients with advanced disease but without pulmonary involvement. This typically includes abdominal and pelvic cross-sectional imaging, along with chest radiography [17].

Due to their hypervascularity, neuroendocrine tumors are best visualized with multiphasic contrast-enhanced computed tomography (CT), particularly during the early arterial and delayed portal venous phases. The diagnostic sensitivity for small lesions can be enhanced by using negative oral contrast agents [20]. Magnetic resonance imaging (MRI) is a suitable alternative in patients with contraindications to iodinated contrast, with hepatic metastases often appearing hyperintense on T2-weighted images [26].

Functional imaging plays a key role in diagnosis, staging, and treatment planning. Radiolabeled somatostatin analogues, such as ^111^In-DTPA-octreotide and ^68^Ga-labeled PET tracers, target somatostatin receptor subtype 2A (SSTR2A), which is highly expressed in well-differentiated NETs. These modalities allow for whole-body imaging and precise localization of lesions [27,28,29,30].

SSTR2A expression also guides the use of somatostatin analogues (SSAs) for symptom control in functional tumors, particularly those causing carcinoid syndrome [31,32,33,34]. Furthermore, it determines eligibility for peptide receptor radionuclide therapy (PRRT), which is a valuable option for advanced or inoperable cases. However, heterogeneous or low receptor expression—especially in poorly differentiated tumors—may limit PRRT efficacy. This variability often correlates with higher Ki-67 indices and remains an area of active research [29].

Fluorodeoxyglucose (FDG) PET-CT, while generally less sensitive for well-differentiated NETs due to low metabolic activity, may be useful in poorly differentiated or non-functioning tumors where FDG uptake is increased [35].

## 8. Biomarkers and Laboratory Assessment

The diagnosis of neuroendocrine tumors (NETs) is confirmed through histopathological and immunohistochemical analysis. However, circulating biomarkers play a complementary role in monitoring disease progression and therapeutic response.

Chromogranin A (CgA) is the most widely used serum marker, elevated in 60–100% of NET patients and generally correlating with tumor burden. Its sensitivity and specificity range from 70% to 100%, although false-positive results may occur, particularly in patients receiving proton pump inhibitors, somatostatin analogues, or those with renal or hepatic dysfunction. For accurate interpretation, CgA should be assessed in relation to therapy timing and after discontinuation of confounding medications [17].

Urinary 5-hydroxyindoleacetic acid (5-HIAA), the primary serotonin metabolite, remains a reliable marker for carcinoid tumors, with approximately 88% specificity. Dietary restrictions—such as avoiding bananas, alcohol, and certain fruits—are necessary before testing to prevent false elevations [36].

Additional biomarkers with supportive diagnostic value include neuron-specific enolase (NSE), pancreastatin, chromogranins B and C, neurotensin, substance P, neurokinin A, and fasting pancreatic polypeptide. Although not routinely used, they may aid in select cases or research settings [17].

## 9. Surgical Management and Oncological Principles

Surgical resection remains the cornerstone of treatment for small intestinal neuroendocrine tumors (NETs), offering both curative and palliative benefits. In a study of approximately 70 small, multifocal tumors, lesions > 1.5 cm were frequently associated with metastases at diagnosis, prompting recommendations for enterectomy based on oncologic principles regardless of metastatic spread [17].

Cholecystectomy is advised during exploration, particularly in patients receiving long-term somatostatin analogues due to an increased risk of cholestasis [36]. Mesenteric lymph node enlargement can result in obstruction, ischemia, or mesenteric fibrosis, but surgical dissection may be technically challenging due to vascular proximity [17].

Symptomatology at presentation is variable. While carcinoid syndrome is rare (<1%) [24], more common manifestations include abdominal pain (36%), facial flushing (26%), and diarrhea (24%) [37]. Some NETs are discovered incidentally during surgery for obstruction. Mesenteric fibrosis, present in up to 50% of cases, may cause significant complications through vascular compromise and local inflammation [38,39].

Histopathologic diagnosis is confirmed by uniform neuroendocrine morphology, “salt and pepper” chromatin, eosinophilic granules suggestive of serotonin content, and diffuse expression of markers such as chromogranin and synaptophysin [40,41].

When feasible, resection of both primary and metastatic lesions within oncologic margins improves survival and symptom control, especially in patients presenting with obstruction, hemorrhage, or ischemia.

## 10. Metastatic Patterns and Prognosis

Although small intestinal neuroendocrine tumors (NETs) are often well-differentiated and slow-growing, they frequently metastasize to the liver, lymph nodes, peritoneum, lungs, and bone [42]. Peritoneal carcinomatosis is observed in up to 14% of well-differentiated cases [43,44], and mesenteric tumor deposits—believed to result from local hematogenous spread—can cause severe complications such as bowel obstruction, ischemia, or ascites [45,46,47,48].

Peritoneal metastases are increasingly recognized as a negative prognostic factor, particularly when associated with mesenteric fibrosis, as illustrated in our case. A pelvic mass consistent with fibrotic mesenteric retraction contributed to obstruction and retroperitoneal extension. Notably, peritoneal spread often coexists with hepatic metastases, further worsening prognosis [49].

Keck et al. found that 72% of primary tumors were located within 100 cm of the ileocecal valve, and over half of patients presented with multifocal disease, justifying systematic palpation during surgery [49]. In a series of 219 patients, peritoneal metastases were found in 67, either intraoperatively or during progression [43,50,51]. Complications such as bowel or ureteral obstruction have been linked to peritoneal carcinomatosis [52].

Despite a low Ki-67 index (3%), our patient’s G2 jejunoileal NET demonstrated aggressive behavior, with widespread hepatic and peritoneal metastases. This disconnect between histologic grade and clinical severity underscores the need for comprehensive staging and multidisciplinary management.

While surgical resection remains central, segmental hepatectomy has not consistently improved survival in metastatic cases [53,54]. Systemic therapies have shown better results for hepatic disease, but effective treatments for peritoneal metastases remain limited. Peritonectomy with intraperitoneal chemotherapy showed potential benefit in one study, though the protocol was discontinued due to toxicity and uncertain efficacy in indolent tumors [43,46].

Survival outcomes remain poor in the long term. In one study, no patients with hepatic metastases survived beyond 20 years [53]. Available treatments include resection, embolization, ablation, and PRRT, particularly in somatostatin receptor-positive tumors [55,56,57]. The presence of peritoneal metastases may also influence staging by suggesting serosal invasion and tumor cell exfoliation, according to Keck et al. [49].

## 11. Guidelines and Current Recommendations

Current guidelines emphasize a multidisciplinary approach for managing metastatic small intestinal neuroendocrine tumors (NETs). According to the European Neuroendocrine Tumor Society (ENETS), surgical resection of hepatic metastases is recommended when feasible, potentially combined with image-guided techniques such as radiofrequency ablation, transarterial chemoembolization, or selective internal radiation therapy.

Peritoneal metastases may also be surgically excised when technically possible. The Canadian and North American guidelines support this approach but do not recommend hyperthermic intraperitoneal chemotherapy (HIPEC), as current evidence fails to demonstrate significant benefit [58].

Hepatic metastases remain the most frequent site of distant spread and often correlate with elevated 24-hour urinary 5-hydroxyindoleacetic acid (5-HIAA) levels, which serve as both diagnostic and prognostic markers in carcinoid syndrome [38].

While liver involvement may limit curative options, resection of the primary tumor can still confer survival benefits. Capurso et al. found that patients undergoing enterectomy—despite unresectable liver metastases—had longer overall survival (75–139 months) compared to those managed non-surgically (50–89 months) [58].

In a Swedish cohort study, early versus delayed locoregional surgery did not significantly impact survival, though delayed intervention was associated with higher reoperation rates [38]. These findings suggest that timely surgical management should be tailored to individual patient profiles.

Despite advances in systemic and locoregional therapies, lymph node metastases may cause mesenteric ischemia or obstruction. Surgical intervention should follow oncologic principles, aiming to preserve bowel function and the ileocecal valve when possible.

## 12. Case Relevance and Specific Insights

This case highlights the diagnostic and therapeutic challenges of small intestinal neuroendocrine tumors (NETs), which often present with vague symptoms and are diagnosed at an advanced stage. Despite a well-differentiated histology (G2, Ki-67: 3%), the patient exhibited aggressive disease features, including peritoneal carcinomatosis, hepatic metastases, ascites, and mesenteric fibrosis. The discrepancy between histologic grade and clinical behavior underscores the limitations of grading alone in predicting prognosis. Comprehensive staging—including advanced imaging, immunohistochemistry, and somatostatin receptor profiling—is essential for accurate assessment and treatment planning.

This case reinforces the importance of early detection, multidisciplinary management, and the potential role of peptide receptor radionuclide therapy (PRRT) in advanced presentations.

## 13. Reflective Commentary

This case underscores several important clinical lessons regarding the management of small intestinal neuroendocrine tumors (SI-NETs), particularly in patients who present acutely with advanced disease. Despite being a well-differentiated tumor with a low Ki-67 index (3%), the patient’s tumor exhibited an unexpectedly aggressive course, with extensive hepatic and peritoneal metastases, as well as significant mesenteric fibrosis resulting in bowel obstruction. This discordance between histological grade and clinical behavior highlights a critical limitation in relying solely on proliferative indices for prognostication.

One of the most notable aspects of this case is the late-stage diagnosis, which reflects a broader issue in the clinical landscape, namely, the difficulty in identifying SI-NETs early due to their often indolent and asymptomatic nature. A greater degree of clinical vigilance is warranted, particularly in patients presenting with nonspecific gastrointestinal symptoms, subtle signs of obstruction, or unexplained mesenteric masses on imaging. Incorporating early functional imaging (e.g., Ga-68 PET/CT) and biomarker assessment (e.g., chromogranin A, 5-HIAA) in suspicious cases may facilitate earlier detection and more effective therapeutic planning.

From a management perspective, the case reinforces the value of prompt surgical intervention in the setting of mechanical obstruction, even in the presence of disseminated disease. Although curative resection was not feasible, surgical palliation provided significant symptom relief and allowed for histological confirmation, thereby guiding further treatment planning, including consideration for peptide receptor radionuclide therapy (PRRT). The role of multidisciplinary care—including surgery, oncology, radiology, pathology, and nuclear medicine—was central to decision making and highlights the need for collaborative, case-by-case strategies in complex NET presentations.

Clinicians should be reminded that well-differentiated morphology does not exclude aggressive clinical progression. This case contributes to the growing body of literature advocating for a more nuanced and integrative approach to SI-NETs—one that balances histopathological grading with functional imaging, tumor burden, receptor profiling, and clinical presentation. Early diagnosis, individualized therapeutic strategies, and long-term multidisciplinary follow-up remain essential components in improving outcomes for patients with advanced neuroendocrine neoplasms.

## 14. Conclusions

This case demonstrates that small intestinal neuroendocrine tumors (NETs) may display aggressive clinical behavior despite low-grade histological features. The presence of extensive peritoneal and hepatic metastases, along with severe mesenteric fibrosis, underscores the limitations of histopathological grading as a sole prognostic tool. This highlights the importance of comprehensive staging strategies that incorporate advanced imaging, immunohistochemistry, and multidisciplinary evaluation, including consideration of peptide receptor radionuclide therapy (PRRT). Early detection and individualized management remain critical to improving outcomes in advanced-stage NETs.

## Data Availability

The data presented in this study are available on request from the corresponding author. The data are not publicly available due to patient confidentiality.
